# Chemical Disinfection of an Accidentally Contaminated and Irreplaceable Inorganic Element During Orthopaedic Surgery Is a Safe Option

**DOI:** 10.2106/JBJS.24.01163

**Published:** 2025-06-19

**Authors:** Jocelyn Corbaz, Dominique S. Blanc, Bruno Grandbastien, Olivier Borens

**Affiliations:** 1 Department of Orthopedics and Traumatology, Lausanne University Hospital, University of Lausanne, Lausanne, Switzerland; 2 Infection Prevention and Control Unit, Infectious Diseases Service, Lausanne University Hospital, University of Lausanne, Lausanne, Switzerland; 3 Bone and Motion Center, Clinique Bois-Cerf, Hirslanden Private Hospital Group, Lausanne, Switzerland

## Abstract

**Background:**

During surgical procedures, the accidental contamination of a critical instrument or implant can jeopardize the entire operation. Resterilizing the item is not always feasible and can be time-consuming. Since extending the duration of the surgery heightens the risk of postoperative complications, it is essential to balance this risk with the risk of infection from contamination. Currently, there is no simple, safe, and quickly available method to address this issue. This study explored the efficacy of using chemical disinfection to deal with this problem.

**Methods:**

In part 1 of the study, 3 types of discs (cobalt-chromium, titanium, and polyethylene) were contaminated with *Staphylococcus epidermidis,* disinfected with use of 3 different procedures (2% chlorhexidine in 70% isopropanol alcohol, 0.9% povidone-iodine in 46% isopropanol alcohol, or 70% ethanol), and analyzed for remaining bacteria. A control group without disinfection was included. In part 2, the discs were dropped on the floor of an operating room, left on the floor for 30 seconds, and then collected before undergoing the same procedure as in part 1.

**Results:**

In part 1, all 3 alcohol-based disinfection procedures showed a high efficacy, as there was no growth found on any of the discs. These results were highly significant compared with those found for the control group (p < 0.01 for all). In the control group, polyethylene had the highest mean level of contamination (157.3 colony-forming units [CFUs]) and titanium had the lowest (58.4 CFUs). Part 2 confirmed the effectiveness of alcohol-based disinfection, with no growth observed in the test cultures. In the control group, polyethylene seemed to be the most prone to contamination. However, the level of contamination was low for all materials (0 to 8 CFUs per disc).

**Conclusions:**

In the event of accidental contamination of an essential element or implant during a surgical procedure with no possibility of replacing the element, 2 minutes of disinfection in an alcohol-based solution seems to be a safe, simple, and quick option.

**Clinical Relevance:**

In the event of accidental contamination of an irreplaceable inorganic element during orthopaedic surgery, we recommend soaking the element in an alcohol-based disinfectant for 2 minutes and rinsing it with saline solution.

The accidental contamination of an essential instrument or implant during a surgical procedure can compromise the intervention if a replacement element is not quickly available. No guidelines exist for procedures to follow in such events, despite many studies showing that accidental contamination secondary to a dropped object is common. Khan et al. reported 39 falls of objects across 120 surgical procedures, with 7 of the falls involving an implant^1^. When considering autografts, 25% of orthopaedic surgeons admit having been confronted at least once with the problem of an accidental contamination^2^, with the rate rising to 70% among plastic surgeons^3^. In most of these cases, the incident was resolved with the use of chemical disinfection in order to avoid a second graft harvest.

In orthopaedic surgery, a postoperative infection can have severe consequences for the patient. Any procedure that affects the chain of sterility raises concerns regarding the safety of such practices. Chemical disinfection of an accidentally contaminated organic element has been studied previously. Studies have evaluated the types of bacteria found on the operating room (OR) floor^4,5^, the number of colonies growing on a contaminated graft versus the number of colonies on a graft that was left on a sterile table in the OR^5^, the risk of implanting an accidentally contaminated graft^5^, and the best disinfectant agent to use in such a situation^4-7^. The conclusions have varied, but recent studies have agreed that relatively low rates of contamination (0.8% to 3%) can be achieved with the use of chlorhexidine^4,6,8^, permitting the implantation of the graft. The decontamination agents evaluated in these studies were those that are usually available in ORs, such as povidone-iodine, chlorhexidine, or a mix of 3 antibiotics^4-6^, and were often followed by the rinsing of the graft in sterile normal saline solution.

All of these studies evaluated the issue of graft contamination, but none addressed the same question with respect to inorganic material, as it is usually easier to simply replace the involved element. However, for essential instruments or implants that cannot be quickly replaced, accidental contamination poses a dilemma regarding the best way to manage the situation. Should the surgery be stopped and a second intervention be planned to finish the procedure once a new sterile element is available? Should the patient wait under anesthesia until the element is available after its sterilization using a normal sterilization procedure or until a replacement element is delivered? Or should the element be cleaned with available decontamination agents, allowing the intervention to be finished without excessive time loss? It is worth noting that a normal sterilization procedure lasts approximately 3 hours, with a full cycle consisting of precleaning, cleaning in a washer or disinfector, and steam sterilization at 134°C for 18 minutes. A quick sterilization process named “flash sterilization”^9^ does exist and lasts approximately 30 minutes but is only applicable to small instruments. Standard sterilization of the contaminated element is often considered to be too time-consuming. In addition to planning issues, a prolonged procedure has been demonstrated to be an independent factor for increased complications (e.g., surgical site infection, deep venous thrombosis, bleeding)^10-12^ and is 1 of the 3 criteria of the National Nosocomial Infections Surveillance System (i.e., wound class, operative duration, and American Society of Anesthesiologists classification)^13^.

We conducted a laboratory study to determine whether chemical disinfection is a safe option in case of accidental contamination of an essential and irreplaceable element involving a prosthesis or instrument during a procedure.

## Materials and Methods

The elements that were utilized were 3 sets of 30 discs (12.7 mm diameter and 3.8 mm thickness, for a total surface area of 405 mm^2^), with each set made of a different material: cobalt-chromium (alloy F1537), polyethylene (ultra-high molecular weight polyethylene), and titanium (alloy Ti-6Al-4V ELI) (BioSurface Technologies) (Figs. 1-A and 1-B). The materials were chosen on the basis of the most used materials for an orthopaedic prosthesis. The discs were sterilized by steam sterilization at 121°C for 20 minutes and packed in a sterile manner. The discs were used a maximum of 4 times each and were cleaned and sterilized before each use. After use, they were dipped into an instrument cleaning agent diluted at 2% (deconex INSTRUMENT PLUS; Borer Chemie) for 15 minutes, then swept and left in tap water until their dispatch to sterilization.

**Fig. 1-A:**
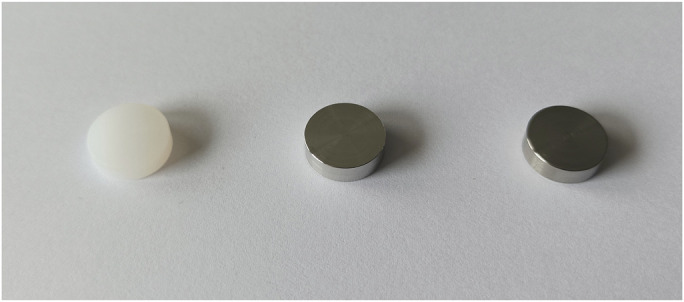
A The discs used for the experiments were made of polyethylene, cobalt-chromium, or titanium.

**Fig. 1-B:**
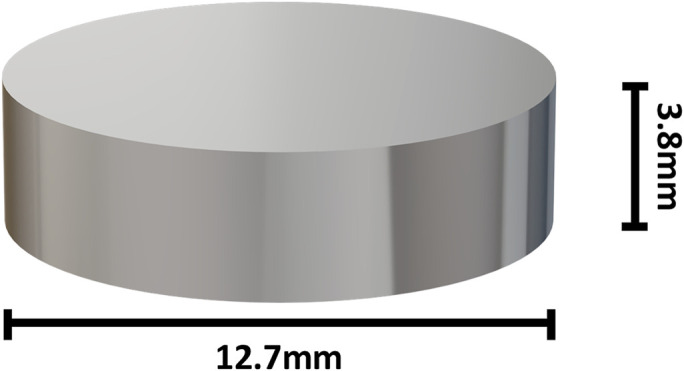
Dimension of the discs.

The study included a preliminary phase and 2 parts involving laboratory tests.

The preliminary phase was aimed at determining the contamination protocol for achieving a level of approximately 100 colony-forming units (CFUs) on a tested disc. Sterile discs were dipped into bacterial suspensions (*Staphylococcus epidermidis* ATCC 12228) of variable concentrations for 10 minutes, then dried on a sterile gauze for 20 minutes under laminar flow. Then, the disc was dipped into 20 mL of peptone water and vortexed for 30 seconds. The suspension was filtered through a 0.45-µm membrane. The membrane was placed on a Columbia culture medium and incubated at 37°C for at least 2 days. The number of CFUs was counted. The choice of using *S. epidermidis* as the contamination strain was based on its human portage, causing its frequent identification in the OR environment^5,14,15^; its ability to create biofilm; and its ability to survive on inert elements. We decided to use a single strain of bacteria in order to facilitate the identification of possible contaminants in the cultures.

**Fig. 2:**
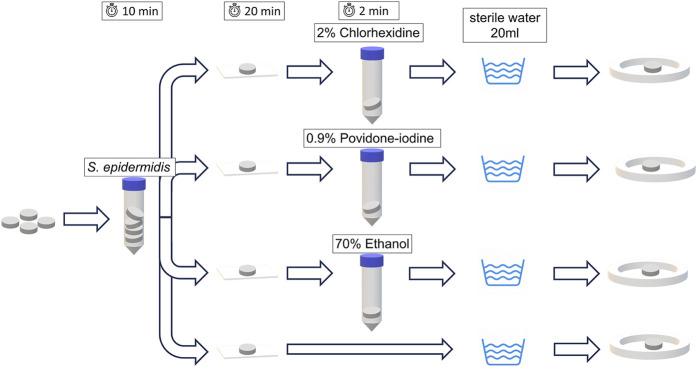
Diagram of the process for part 1. Discs were artificially contaminated with *S. epidermidis* for 10 minutes, then left to dry on a sterile gauze for 20 minutes. Afterward, they were disinfected with 3 different disinfectants, except in 1 control group, which did not undergo disinfection. All discs were then rinsed with 20 mL of sterile water and cultured.

Following the preliminary phase, part 1 of the study was aimed at evaluating the efficacy of chemical disinfection of an intentionally contaminated element (Fig. 2). Each disc was contaminated by soaking it for 10 minutes in a bacterial suspension of *S. epidermidis* at the concentration determined during the preliminary phase. The discs were then left for 20 minutes on a dry and sterile gauze under laminar flow in order to worsen the situation, giving the bacteria some time to adhere to the surface. Afterward, they were separated into 4 groups: 1 control group, and 3 groups undergoing disinfection for 2 minutes in 3 different disinfectants. The 3 disinfectants that were used were (1) 2% chlorhexidine in 70% isopropanol alcohol (Chlorhexidine 2% alcoholic solution uncoloured; B. Braun Medical), (2) 0.9% povidone-iodine in 46% isopropanol alcohol (Braunoderm alcoholic solution coloured; B. Braun Medical), and (3) 70% ethanol. After disinfection, the discs were rinsed in 20 mL of sterile water to remove residual disinfectant left on the discs, which could hinder the growth of any surviving bacteria on the culture medium. Thereafter, they were cultured using the same standardized procedure as in the preliminary experiments. The control group underwent no disinfection and was only rinsed in 20 mL of sterile water and cultured using the same standardized procedure. Each test was conducted 10 times for each different material and each disinfectant.

Part 2 of the study was aimed at testing the disinfection protocol in a “close to real-life” situation. The discs were dropped on the floor of an OR at the end of an orthopaedic intervention, left on the floor for 30 seconds, and then collected with use of sterile gloves before undergoing the same procedure as in part 1. Ten discs of each of the 3 materials were tested for each disinfectant and for the control group.

Results are expressed as the average of the CFUs observed across the 10 iterations. For the statistical analyses, an unpaired Student t test was used to calculate the p values. A p value of <0.05 was considered significant. The confidence interval (CI) was set at 95%.

## Results

### Preliminary Experiments

The number of CFUs recovered from the 3 different discs after the discs were dipped into different concentrations of *S. epidermidis* suspension is displayed in Table 1. A bacterial suspension of 10^7^ CFU/mL contaminated the disc at a rate of approximately 150 to 500 CFUs per disc, which was the level of contamination desired. This suspension of bacteria was used for the experiment in part 1.

**Table 1.** Number of CFUs on Discs After Contamination in *S. epidermidis* Suspension at Different Concentration Levels*

Disc Type	Concentration Level of *S. epidermidis* Suspension		
	10^8^ CFU/mL	10^7^ CFU/mL	10^6^ CFU/mL
Polyethylene	>400	471	70
Cobalt-chromium	>400	511	31
Titanium	>400	159	4

* Values are given as the mean number of CFUs per disc.

### Part 1

The 3 types of discs were dipped into a bacterial suspension of 10^7^ CFU/mL, disinfected with the 3 different agents, and analyzed for residual contamination. The experiment was repeated 10 times. After soaking the contaminated discs in the alcohol-based disinfectant for 2 minutes, no growth of bacteria was found, regardless of the type of alcohol-based disinfectant (chlorhexidine, povidone-iodine, or ethanol) and the type of disc (Table 2). These results were highly significant when compared with those in the control group, with mean differences [MDs] of 157 CFUs (95% CI, 73 to ∞; p = 0.002) for polyethylene, 70 CFUs (95% CI, 42 to ∞; p < 0.001) for cobalt-chromium, and 58 CFUs (95% CI, 23 to ∞; p = 0.007) for titanium. The control group was found to be contaminated at the predicted level, as calculated in the preliminary experiments.

**Table 2.** Number of CFUs (Mean of 10 Trials) After Disinfection of Discs Contaminated in *S. epidermidis* Suspension

Disc Type	Mean CFU/Disc				95% CI of Mean Difference (Control Vs. Non-Controls)	P Value
	Chlorhexidine	Povidone-Iodine	Ethanol	Control*		
Polyethylene	0	0	0	157	73 to ∞	0.002
Cobalt-chromium	0	0	0	70	42 to ∞	<0.001
Titanium	0	0	0	58	23 to ∞	0.007

* The control group did not undergo disinfection.

In the control group, polyethylene had the highest mean level of contamination (157.3 CFUs), titanium had the lowest (58.4 CFUs), and cobalt-chromium was in between (69.8 CFUs). This difference was close to reaching significance when comparing polyethylene and titanium (MD, 99 CFUs; 95% CI, −6 to 203; p = 0.06) but did not reach significance when comparing cobalt-chromium and titanium (MD, 11 CFUs; 95% CI, −40 to 63; p = 0.65) or polyethylene and cobalt-chromium (MD, 88 CFUs; 95% CI, −14 to 189; p = 0.09).

Nonparametric analyses using the Mann-Whitney U test were also performed and gave similar results.

### Part 2

The discs that were dropped in the OR showed no bacterial growth after chemical disinfection (Table 3). In the control group, no growth was observed in 26 of 30 experiments, and 1 to 8 CFUs were observed in the remaining 4 experiments. Identification of the growing bacteria showed that the majority belonged to human flora (*S. epidermidis, Staphylococcus warneri, Staphylococcus hominis, Staphylococcus borealis, Kocuria palustris, Corynebacterium afermentans,* and *Corynebacterium tuberculostearicum*).

**Table 3.** Number of CFUs (Mean of 10 Trials) After Disinfection of Discs Dropped on the OR Floor

Disc Type	Mean CFU/Disc				95% CI of Mean Difference (Control Vs. Non-Controls)	P Value
	Chlorhexidine	Povidone-Iodine	Ethanol	Control*		
Polyethylene	0	0	0	1.5	−0.12 to ∞	0.06
Cobalt-chromium	0	0	0	0	0 to 0	–
Titanium	0	0	0	0.6	−0.5 to ∞	0.17

* The control group did not undergo disinfection.

Polyethylene seemed to be the material most susceptible to contamination when dropped on the floor, similar to the findings from part 1.

## Discussion

To our knowledge, no other study has examined the use of a chemical disinfectant on inorganic material as a possible solution to the accidental contamination of an essential and not easily replaceable element during orthopaedic surgery. Previous studies were performed on grafts with non-alcohol-based solutions^4-8^. Most of the accidental contaminations in ORs can be managed by replacing the involved element, and this solution should always be favored since surgical instruments and implants have to follow a very precise and well-established procedure of sterilization for patient-safety reasons. From time to time, however, this may not be possible or may be at the cost of a long wait, which can increase the duration of surgery and the risks of complications for the patient^10-12^.

The present study showed that a simple procedure of disinfection with an alcohol-based disinfectant can be very effective, with no residual bacterial growth found after disinfection of elements that were either artificially heavily contaminated (part 1) or contaminated after being dropped on an OR floor (part 2). The level of CFUs in the control group during part 1 proved that the artificial contamination was effective and at the expected level (around 100 CFUs). Additionally, the level of contamination in the control group in part 1 (range, 58 to 157 CFUs) was much higher than that found in part 2 (range, 0 to 8 CFUs), confirming that the artificial contamination (part 1) mimicked a very heavy contamination. The use of a “worst-case scenario” (heavy contamination; the use of a strain capable of adhering to, and surviving on, an inert surface; waiting 20 minutes before dipping the discs in disinfectant solutions) during the laboratory tests validates the efficacy of the disinfection for the lower level of contamination encountered in a real-life OR environment. The level of contamination in the control group in part 2 was surprisingly low, but studies on the contamination level of the OR floor are scarce. Balkissoon et al.^16^ also found low CFU levels on OR floors and similar germs (mainly Staphylococcus spp. and Corynebacterium spp.).

In the event of accidental contamination of an essential element or implant during a surgical procedure with no possibility of replacing the element, 2 minutes of disinfection in an alcohol-based solution seems to be a safe, simple, and quick option.

Our study has limitations. As in any in vitro study, only suggestions can be made regarding the in vivo situation. The ideal study would be an in vivo comparative study with a control group, but such a study could hardly be justified from an ethical point of view, even more so in light of the present results. An observational study could be imagined, but, as such accidental contaminations are rare and the rate of expected infection after disinfection is very low, the number of patients required for such a study would need to be enormous to reach a level of power sufficient to produce significant results. Alcohol has a limited effect on mycobacteria and spores^17^, which may be a source of infection following the implantation of a chemically disinfected element in a patient. Povidone-iodine, with its fungicidal and sporicidal properties—properties that chlorhexidine does not have—would therefore be the adjunct of choice^17^. The surfaces of the discs utilized in the present study were smooth; therefore, a porous-coated surface, as in a press-fit prosthesis, may be more difficult to disinfect using the proposed protocol. The effect of alcohol-based disinfectants, especially on polyethylene, is unknown, and thus the possibility of some alteration of the component cannot be excluded. When using alcohol-based solutions, the fire hazard with electrocoagulation should be kept in mind. The discs were reused up to 4 times, undergoing cleaning and sterilization between each use. These procedures may have altered the surfaces of the discs, especially for polyethylene.

### Conclusions

Our study showed that a simple and quick procedure of chemical disinfection with alcohol-based disinfectants that are available in all ORs could be a safe option in case of accidental contamination of an essential and not easily replaceable element during an orthopaedic procedure. This technique has the advantages of having a very short duration of execution (2 minutes), avoiding any substantial increase in surgical time and anesthetic risk, and not requiring any special equipment or complicated procedure that would limit its use. Therefore, when faced with a situation during a surgical procedure where an essential element has been accidentally contaminated, with no easy replacement available, we recommend soaking the element in an alcohol-based disinfectant agent for 2 minutes and then thoroughly washing it with sterile saline solution, drying it, and using it as intended. Nevertheless, the replacement of the contaminated element, if possible, should always be favored, given that alcohol-based disinfectants have a limited effect on some pathogens (especially mycobacteria and spores) and the consequences of a postoperative infection can be devastating and irreversible, especially in immunocompromised patients^10-12^.
